# Supplementary material for: PreprintMatch: A tool for preprint to publication detection shows global inequities in scientific publication
Source: PLoS One. 2023 Mar 8;18(3):e0281659. doi: 10.1371/journal.pone.0281659 (PMC9994746; doi:10.1371/journal.pone.0281659)
Supplement: Attachment — Submitted filename: COMMENTS FOR THE AUTHOR-PONE-D-22-23440.pdf [file pone.0281659.s002.pdf]

The paper presents a new tool, PreprintMatch, developed by the authors for matching preprints and papers with high efficiency and accuracy, and compare the tool to other existing techniques (e.g., SAGE Rejected Articles Tracker and Cabanac et al.'s tool). With the matches found by PreprintMatch, the authors explored questions related to research inequity at the country level, in particular, looks at country income as a factor, and in some degree, provides quantitative evidence for the issue that why lower income countries produce less papers than high income countries. As a whole, results are supportive of a positive function of preprints in democratizing scientific publishing.

The paper reads well and is very interesting. The methods used in the study is technically sound and the PreprintMatch description provides a sufficient amount of data and information for readers and other researchers to understand the technologies and recreate the analyses. The analyses are well crafted and in general, the interpretations of results are adequate. I suggest the paper for publication after minor revisions.

**1. Causality vs. association.** In the Inequity analysis section, the authors categorize countries into three income groups for analyzing preprint publishing on the country level. Overall, the results are interesting and inspiring, though it is unclear to me whether the authors simply aim to provide descriptive evidence, or are rather arguing on causality. For example, the first paragraph on P.19 "Preprints with collaborations with high income countries were published as papers at a significantly higher rate (52.7%) than preprints without such collaborations, suggesting that collaboration with high income countries is beneficial, in terms of publications, to researchers from lower income countries.", imply a causal relationship between preprint publishing and a country's income level, but, in my opinion, the analysis methods applied in the study cannot be able to go into causal discussion, since the data are only available as aggregated statistics, which cannot be assigned to individual users.

Besides, the time factor, in my opinion, is a very important factor that would affect the results of preprint publishing behavior on the country level, especially for countries not in the high income group. Thus, I am interested in further comparative analysis from the time dimension, to see changes among the three income groups,

**2. Contribution.** The paper provides its contributions (on P.3, the last paragraph in Introduction section) to developing the new tool, PrewprintMatch. I recommend the authors add the paper's contribution on exploring the issue of research inequity.

**3. The descriptions of Fig 5.b and Fig 5.c should be swapped.** That is "(b) Percentage of preprints published from upper middle or low/lower middle income countries where there is at least one other author on the preprint from a high income country or not. (c) Percentage of authors who retain their position from preprint to paper for each income group and for first and last author positions."
